# An Examination of New Paradigms for Spline Approximations

**DOI:** 10.6028/jres.111.005

**Published:** 2006-04-01

**Authors:** Christoph Witzgall, David E. Gilsinn, Marjorie A. McClain

**Affiliations:** National Institute of Standards and Technology, Gaithersburg, MD 20899-8910

**Keywords:** bivariate splines, curve fitting, Delaunay triangulation, Gauss-Seidel iteration, Hsieh-Clough-Tocher elements, irregular data, Lavery splines, non-oscillatory splines, point clouds, surface fitting, thin beam, thin plate, triangulated irregular networks

## Abstract

Lavery splines are examined in the univariate and bivariate cases. In both instances relaxation based algorithms for approximate calculation of Lavery splines are proposed. Following previous work Gilsinn, et al. [7] addressing the bivariate case, a rotationally invariant functional is assumed. The version of bivariate splines proposed in this paper also aims at irregularly spaced data and uses Hseih-Clough-Tocher elements based on the triangulated irregular network (TIN) concept. In this paper, the univariate case, however, is investigated in greater detail so as to further the understanding of the bivariate case.

## 1. Introduction

**Fig. 1:**
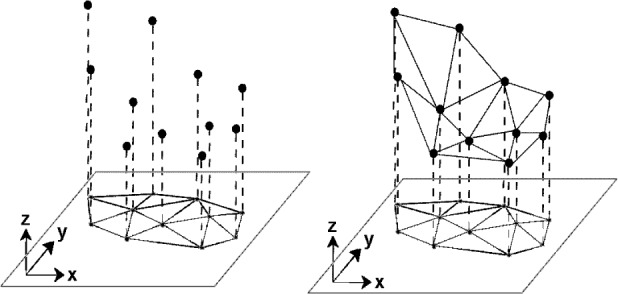
TIN meshing: Triangulated surface over a triangulation in the footprint plane.

**Fig. 2:**
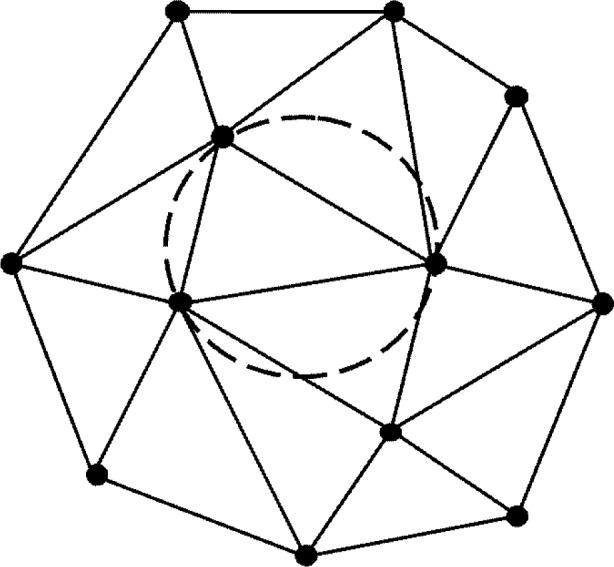
Delaunay triangulation: Circumcircles of triangles do not contain vertices in their interior.

**Fig. 3:**
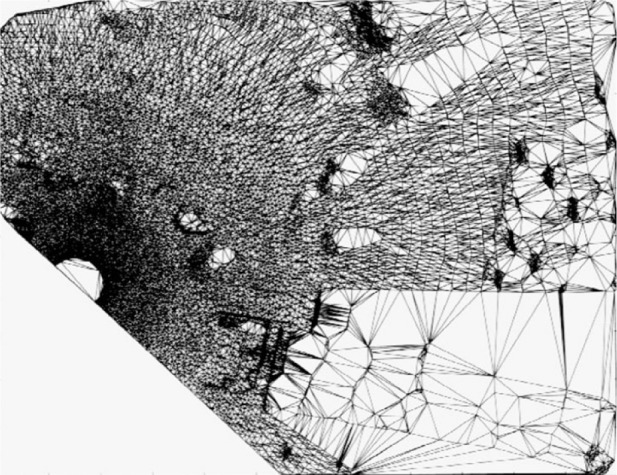
A TIN created from the LADAR scan of terrain.

Over the last decade the task of representing very large “point clouds” by meshed surfaces has arisen in many applications. Such point clouds may have been generated from terrain surveys or from LADAR (**LA**ser **D**irection **A**nd **R**anging) scans of objects. Work has centered around the concept and application of **T**riangulated **I**rregular **N**etwork (TIN). Here the “foot prints” (*x_i_*, *y_i_*) of data points (*x_i_*, *y_i_*, *z_i_*) are “triangulated”, that is, the area of interest is tiled by non-overlapping triangles defining a piecewise triangular “TIN-surface” in space as each triangle is lifted according to the elevations *z_i_* at its corners as indicated in Fig. 1. Most often, the triangulation used is the “Delaunay” triangulation, characterized by the “empty circle property” in which the circumcircle of each triangle does not contain any triangle corners in its interior (see Fig. 2). Figure 3 features such a Delaunay triangulation, underlying a TIN, created from a LADAR scan of a construction site on NIST grounds. The construction of the surface from adjacent patches or “elements” is basically a “Finite Element” technique.

TIN constructs utilize the actual data points directly even if they are distributed irregularly rather than referring to approximations by regular rectangular grids. They adapt naturally to disparate data densities. Importantly, they support strategic selection of data points to be used as the support points for a TIN surface.

Conceptually, TINs define which points may be considered “neighbors” of each other. This proximity concept based on a direct neighbor criterion differs from concepts based on a fixed distance cutoff in that it adjusts automatically to differing data densities such as in data collected by ground based LADAR. Figure 3 exemplifies the dramatic differences in densities encountered in ground-based LADAR scans. Also, because of the neighbor relation provided by a TIN, the utility provided by that construct is not restricted to surface generation, but extends to data editing and analysis.

The TIN surface, as defined according to Fig. 1, is *C*^0^, that is, it is continuous but usually not smooth: it is typically not differentiable along triangle edges, since the plane of any of the spatial triangles constituting the surface generally differs from the planes of its adjacent triangles. The TIN concept of triangulation-based surface generation is frequently understood to imply the use of such planar “elements” resulting in a non-smooth, piecewise linear TIN surface. It should be emphasized, however, that the TIN concept also supports the use of non planar, that is, curved elements for a smooth or *C*^1^ surface. Planar elements offer some advantages besides simplicity and a certain robustness to be discussed below. Visualization still requires piecewise linear representations in order to delineate hidden surfaces. Volume computations similarly tend to be either grid-based or based on piecewise linear surfaces for ease of computation. In those cases, a smooth surface would have to be discretized or approximated by a piecewise linear surface. This then begs the question, why not use planar elements in the first place?

Without any doubt, however, there are many instances, in which a smooth surface representation would provide definite advantages:

- More accuracy could be achieved without increasing memory requirements because curved elements encode more geometric information than planar elements.
- Clusters of coplanar points are avoided when sampling the surface at a discrete set of foot prints such as regular grid points.
- When simulating movement over a terrain surface, a smooth ride is preferable. A ride over a piecewise linear surface is necessarily bumpy at transitions from one triangle to another.
- In many applications, several point clouds which were collected with reference to different coordinate systems need to be “registered”, that is, combined within a common coordinate system. Some common registration methods, such as point-to-surface iterative closest point (ICP) proposed by Besl [2], require minimizing the deviations of points in one point cloud from the surface representation of another point cloud. This minimization process should work much better if that surface were smooth.

In computer aided design (CAD), design-defined objects are routinely represented by surfaces that are *C*^1^ or even *C*^2^. Terrain representation, however, poses a different challenge: The location of “crease lines” or “break lines”, along which the actual terrain surfaces are not differentiable, are usually not known ahead of time, whereas in a CAD environment break lines tend to be specified as part of the design. Unspecified break lines, on the other hand, along with actual verticalities, tend to give rise to spurious oscillation and, what may be called, “Gibbs phenomena” in analogy to the phenomenon well known from the theory of Fourier series. TIN surfaces based on planar elements, on the other hand, are more robust, and can be constructed so as to automatically represent and, if necessary, report break lines. Susceptibility to spurious oscillations and Gibbs phenomena are one of the reasons why the terrain modeling community has been slow to accept smooth surfaces. There has thus been a long quest for “non-oscillatory splines” which would obviate this pesky conundrum.

In 1994, Lavery [11,12,13] (see also Gilsinn and Lavery [8,9,10]) proposed successful paradigms for univariate as well as bivariate non-oscillatory splines, which could be used for representing 2D or 3D data sets, respectively. Lavery introduced the term “*L*_1_ splines” for his brand of nonoscillatory splines. The term *L*_1_ splines is, however, frequently misinterpreted as minimizing an *L*_1_ measure-of-fit when approximating a point set by, say, classical splines. For this reason, we prefer the term “Lavery splines”.

Classical splines are characterized by their minimizing energy functionals. Lavery splines, on the other hand, minimize different functionals. In the bivariate case, especially, the computational effort of minimizing these functionals, however, exceeds the effort required by the classical approach by an order of magnitude. For the bivariate case we have, therefore, considered an approximation to the calculation of Lavery splines, along with a prior modification of the functional proposed by Lavery for the bivariate case. This modified bivariate functional is still an extension of Lavery’s functional for univariate functions, but it extends a different aspect of the latter. It also is invariant under planar rotations of the coordinate system, which Lavery’s bivariate functional is not. We will present preliminary results for both univariate and bivariate nonoscillatory splines.

The main thrust of this paper, however, remains to illustrate the utility and performance of bivariate non-oscillatory splines, building on the previous study by Gilsinn et al. [7], and also on an early terrain modeling study by Mandel et al. [18]. Both studies employ TIN techniques in conjunction with the “reduced Hsieh-Clough-Tocher (rHCT)” element, the approach was pioneered by Lawson [14,15] and is followed in this work. It involves specifying elevations *z_i_* and two slopes *z_ix_*, *z_iy_* at each triangle corner or vertex *v_i_* = (*x_i_*, *y_i_*) in a triangulation, and filling in, at each triangle, the thereby defined rHCT elements, results in a smooth surface over an entire TIN.

The classical spline approach to, say, interpolating the elevations at vertices of a triangulation would be to prescribe the elevations *z_i_*, and select the remaining parameters, namely the partial slopes *z_ix_*, *z_iy_*, so as to minimize some energy functional. This was indeed the approach taken in the work by Mandel et al. [18] The task to be accomplished there was to represent terrain given by digitized contour lines. The elevations at the data points were thus determined by the elevation of the contour line on which the data point was located. In the end, elevations were to be evaluated at the points of a square 900 × 900 grid. At the time, — 1985 — visualization procedures that are now routine were not yet commonly available. A display of the results had to wait another year. So assessing the quality of the representation was restricted to manual spot checking. It thus took until a day before a scheduled presentation that, to our dismay, huge oscillations were detected. Fortunately, it turned out that it was not due to a problem with the method but was caused by an error in the data that had been provided: the elevation of a single contour line had been tagged 100 feet too high, causing the disruption. In this instance, the oscillations proved to be actually beneficial in that they uncovered data errors.

In Sec. 2 we discuss aspects of univariate Lavery splines in order to shed light on related issues for bivariate splines, which are addressed in Sec. 3. The algorithm proposed here for bivariate non-oscillatory splines requires solving large sparse systems of linear equations. Experience was gathered concerning the performance of the well known Gauss-Seidel algorithm.

## 2. Univariate Spline Interpolation

While the emphasis of this work is on bivariate splines for surface generation, an examination of univariate splines is taken up in order to highlight some of the issues pertaining to splines in general. In the univariate case, cubic “spline functions” are most commonly used and are considered here. They form a linear space

F

of piecewise cubic *C*^1^ functions *f* (*x*) defined locally over intervals between “knots”

$$x_{0}<x_{1}<\ldots <x_{n},$$

that is, they consist of cubic polynomials

$$f_{i}(x),x\in [x_{i-1},x_{i}],i=1,\ldots n.$$

Adjacent cubic polynomials are required to assume the same values *y_i_* at common interior knots,

$$y_{i}=f_{i}(x)=f_{i+1}(x_{i}).$$

This ensures continuity of the complete spline function *f* (*x*) over the entire interval

$$I=[x_{0},x_{n}].$$

In addition, the polynomials are to assume the same slopes

$$m_{i}=f_{i}^{\prime }(x_{i})=f_{i+1}^{\prime }(x_{i}).$$

The spline functions *f* (*x*) are thus continuously differentiable, that is, they belong to class *C*^1^. In what follows, the linear spaces

$$F^{\prime },F^{\prime \prime }$$

of first and second derivatives of spline functions are also considered, in spite of the fact that, at common knots, the second derivatives of adjacent cubic polynomials may not agree, so that the spline functions *f* (*x*) ∈ *F* are generally not twice differentiable at such knots. However, they are twice differentiable everywhere but on this set of measure zero. For the purposes of integration below, it does not matter that the function *f*″(*x*) may not be defined for those arguments.

**Fig. 4:**
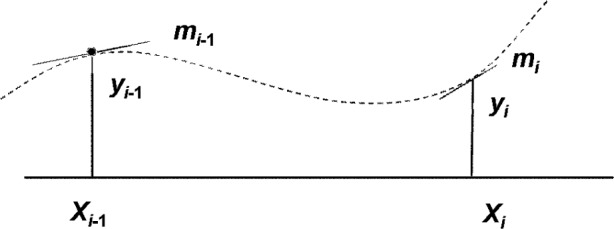
Hermite cubic polynomial determined by coordinates and slopes at end points.

Each of the constituent cubic polynomials *f_i_*(*x*) is uniquely determined by the values *y_i_*_−1_, *y_i_* at the knots *x_i_*_−1_, *x_i_* and the slopes *m_i_*_−1_, *m_i_* at those locations (Fig. 4), in fact, the polynomial is linear in these parameters. The entire function *f* (*x*) is thus uniquely determined by its values and slopes at the knots, and it, too, depends linearly on these parameters, so that the space *F* is isomorphic to the 2(*n*+1)-dimensional vector space of values *y_i_* and slopes *m_i_*, *i* = 0, …, *n*.

We now turn to the task of interpolation. Here the values *y_i_* at the knots *x_i_* are fixed and specified. Given a particular specification of values *y_i_*, a corresponding “interpolating spline function” depends only on the parameters *m_i_*:

$$f(x)=f(m_{0},m_{1},\ldots ,m_{n};x).$$

Collectively, these functions define affine manifolds or flats,

$$S,S^{\prime },S^{\prime \prime },$$

within the linear spaces *F*, *F*′, *F*″, respectively. That is, if

$$m_{i}=\frac{m_{i}^{\left(1\right)}+m_{i}^{\left(2\right)}}{2},i=0,1,\ldots n,$$

then it follows that correspondingly

$$\begin{array}{l} f(x)=f(m_{0},m_{1},\ldots m_{n};x)\\ \;=\frac{f(m_{0}^{\left(1\right)},m_{1}^{\left(1\right)},\ldots ,m_{n}^{\left(1\right)};x)+f(m_{0}^{\left(2\right)},m_{1}^{\left(2\right)},\ldots ,m_{n}^{\left(2\right)};x)}{2}. \end{array}$$

The question then becomes, how to select slopes *m_i_* so as to achieve a “satisfactory” interpolation. That selection is generally made by minimizing a functional on the affine space *S* defined by an integral. In this work, we reserve the term “spline” — as opposed to “spline function”, for the results of such a minimization.

### 2.1 Paradigms for Univariate Splines

“Classical splines” are uniquely defined as those interpolating cubic spline functions which are (i) *C*^2^, that is, twice differentiable, such that

$$f_{i}^{\prime \prime }(x_{i})={f_{i+1}}^{\prime \prime }(x_{i}),i=1,\ldots ,n-1$$ (1)

holds at interior knots, and for which (ii) the second derivative vanishes

$$f^{\prime \prime }(x_{0})=f^{\prime \prime }(x_{n})=0$$ (2)

at the two exterior knots. Holladay proved early on (see Ahlberg [1]) that classical splines are also defined as the unique minimizers of the “thin beam energy”

$$E(f)=\int_{x_{0}}^{x_{n}}f^{\prime \prime }{\left(x\right)}^{2}dx$$ (3)

over the affine space *S* of all *C*^1^ interpolating spline functions.

Condition (2) is familiar to structural engineers as the vanishing of the second derivative at the “free end” of a beam. It is, however, remarkable that this energy minimization enforces also a higher level of compatibility across knots (1) so that the minimizing *C*^1^ spline functions are, in fact, in class *C*^2^. On the other hand, this stiffness contributes to the tendency of classical splines to produce spurious oscillations, Gibbs phenomena and undesirable inflections.

Several attempts have been made to avoid these problems. Taking a clue from mechanics, Schweikert [20] and Cline [3] have introduced “tension splines”, where the arclength of the spline function is made part of the defining minimization. Reinsch (see Stoer and Bulirsch [21]) moved to “exponential splines” by adding a multiple of the square of first derivatives to the integrand in (3). Those efforts were only partially successful. Drawbacks include the need to specify an additional parameter in order to balance conflicting minimization requirements, and the fact that these techniques are not readily generalized to the bivariate case.

**Fig. 5:**
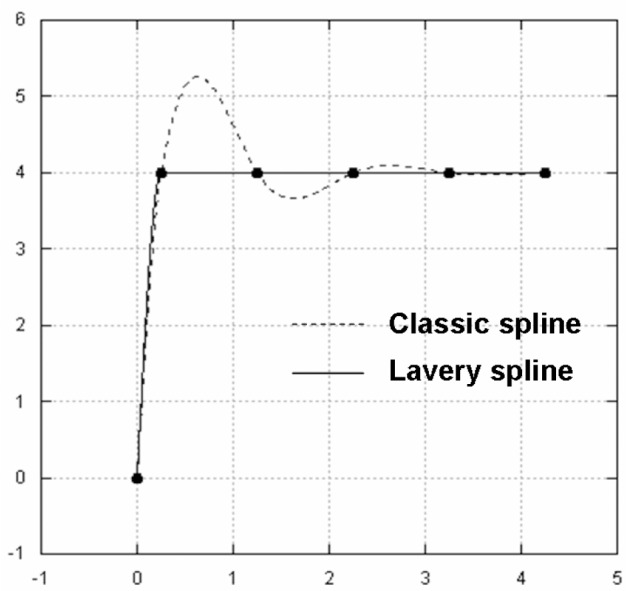
Lavery spline vs. classic spline: Example 1.

**Fig. 6:**
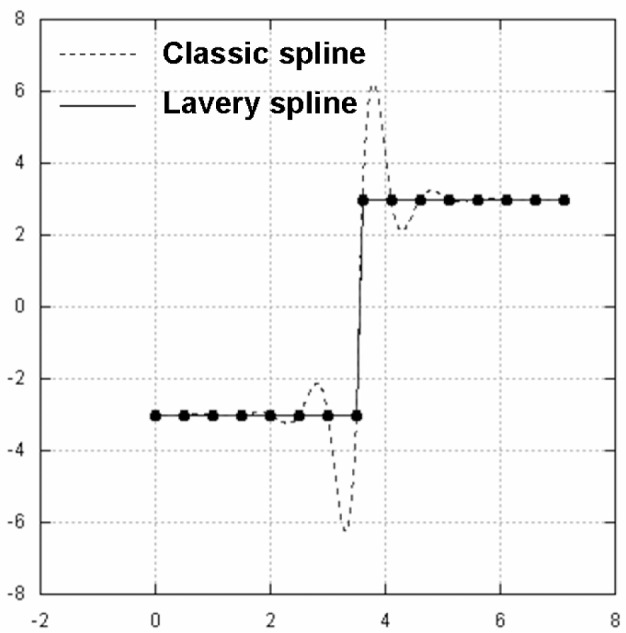
Lavery spline vs. classic spline: Example 2.

**Fig. 7:**
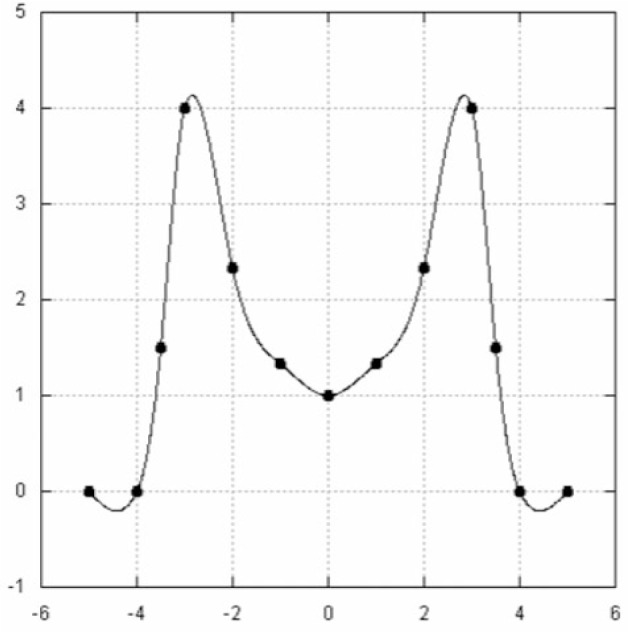
Classic splines produce unnecessary variations in curvature.

**Fig. 8:**
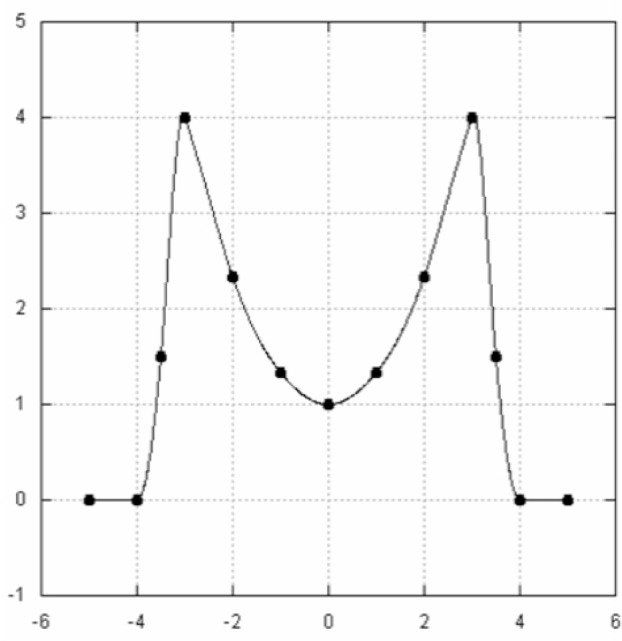
Lavery spline has smooth curvature transitions.

Lavery splines, on the other hand, appear to avoid such shortcomings. In Figs. 5 and 6 we compare Lavery splines against classic splines. We are particularly impressed with the performance of Lavery splines for the example in Fig. 8, as compared to the example in Fig. 7. What is the secret of Lavery splines? How are they defined as opposed to classical splines?

Lavery defines his nonoscillatory splines, in essence, as minimizing the integral of the absolute value rather than the square of the second derivative of a spline function:

$$e(f)=\int_{x_{0}}^{x_{n}}|f^{\prime \prime }(x)|dx$$ (4)

over the given affine space *S* of interpolating spline functions.

### 2.2 Expressing Holladay and Lavery Integrals

For the purposes of this paper, we will refer to the integrals (3) and (4) as the “Holladay integral” and the “Lavery integral”, respectively. The goal of this section is to derive expressions for the values of these integrals in terms of the slopes *m_i_*, *i* = 1, …, *n*, at the knots of the spline function *f* (*x*) under consideration. Both are the sums of the corresponding integrals of the second derivatives of the individual cubic polynomials *f_i_*(*x*), which constitute the spline function *f* (*x*).

As pointed out in Sec. 2.1, each such cubic polynomial is uniquely defined by its end points (*x_i_*_−1_, *y_i_*_−1_), (*x_i_*, *y_i_*) and its end slopes *m_i_*_−1_, *m_i_*. The various formulas describing this polynomial are commonly referred to as “Hermite” formulas. For the versions used here, we introduce the quantities

$$\Delta_{i}=x_{i}-x_{i-1}$$

and

$$M_{i}=\frac{y_{i}-y_{i-1}}{x_{i}-x_{i-1}}=\frac{y_{i}-y_{i-1}}{\Delta_{i}},$$

where *M_i_* represents the slope of the straight line between end points. Instead of referring to the variable *x* directly, the following formulas are in terms of the weights

$$\begin{array}{l} \lambda_{i}=\lambda_{i}(x)=\frac{x_{i}-x}{x_{i}-x_{i-1}}=\frac{x_{i}-x}{\Delta_{i}},\\ \mu_{i}=\mu_{i}(x)=\frac{x_{i}-x_{i-1}}{x_{i}-x_{i-1}}=\frac{x-x_{i-1}}{\Delta_{i}}, \end{array}$$ (5)

where *λ_i_* + *µ_i_* = 1, and *λ_i_*, *µ_i_* ≥ 0, for *x* in the interval [*x_i_*_−1_, *x_i_*]. Such weights are often referred to as “barycentric coordinates” or, in the bivariate case, as “triangle coordinates”. With these conventions, we find, for instance,

$$f_{i}(x)=\lambda_{i}y_{i-1}+\mu_{i}y_{i}+\lambda_{i}^{2}\mu_{i}(m_{i-1}-M_{i})\Delta_{i}-\lambda_{i}\mu_{i}^{2}(m_{i}-M_{i})\Delta_{i}.$$ (6)

Furthermore, by definition (5),

$$dx=-\Delta_{i}d\lambda_{i}=+\Delta_{i}d\mu_{i},$$

the chain rule yields, using (*λ_i_* + *µ_i_*)^2^ = 1,

$$f_{i}^{\prime }(x)=6\lambda_{i}\mu_{i}M_{i}+(\lambda_{i}^{2}-2\lambda_{i}\mu_{i})m_{i-1}+(\mu_{i}^{2}-2\lambda_{i}\mu_{i})m_{i}.$$

An alternate expression for the first derivative is readily derived:

$$f_{i}^{\prime }(x)=\lambda_{i}m_{i-1}+\mu_{i}m_{i}+\lambda_{i}\mu_{i}D_{i}.$$ (7)

Here the quantity

$$D_{i}=6M_{i}-3m_{i-1}-3m_{i}=6\left(M_{i}-\frac{m_{i-1}+m_{i}}{2}\right),$$ (8)

vanishes if and only if the polynomial *f* (*x*) has degree less than three. Because by (5)

$$\lambda_{i}\mu_{i}=\left(\frac{x_{i}-x}{\Delta_{i}}\right)\left(\frac{x-x_{i-1}}{\Delta_{i}}\right)$$

−*D_i_* is seen as the lead coefficient of $f_{i}^{\prime }(x)$ as expressed in *x*. Consequently −1/3*D_i_* is the lead coefficient of *f_i_*(*x*). *D_i_* < 0 indicates that the function is concave up to its inflection point and convex thereafter. Conversely, *D_i_* > 0 indicates that convexity precedes concavity in the direction of the *x*-axis. The quantity *D_i_* will play a major role in what follows. The same is true for the quantities *U_i_*, *V_i_* in the expression

$$f_{i}^{\prime \prime }(x)=\frac{2}{\Delta_{i}}(\lambda_{i}U_{i}-\mu_{i}V_{i}),$$ (9)

where

$$\begin{array}{l} U_{i}=3M_{i}-2m_{i-1}-m_{i}=+\frac{\Delta_{i}}{2}f_{i}^{\prime \prime }(x_{i-1})\\ V_{i}=3M_{i}-m_{i-1}-2m_{i}=-\frac{\Delta_{i}}{2}f_{i}^{\prime \prime }(x_{i}). \end{array}$$ (10)

The coefficients *U_i_*, *V_i_* thus relate to the two-sided second derivatives of *f_i_*(*x*) at knots *x_i_*. Note that

$$D_{i}=U_{i}+V_{i}$$

and also that, in view of (5),

$$\int_{x_{i-1}}^{x_{i}}\lambda_{i}^{2}dx=\frac{\Delta_{i}}{3},\int_{x_{i-1}}^{x_{i}}\lambda_{i}\mu_{i}dx=\frac{\Delta_{i}}{6},\int_{x_{i-1}}^{x_{i}}\mu_{i}^{2}dx=\frac{\Delta_{i}}{3}.$$

An expression for the thin beam energy of the individual polynomial *f_i_*(*x*) is now readily derived:

$$E(f_{i})=\frac{4}{\Delta_{i}}[m_{i-1}^{2}+m_{i-1}m_{i}+m_{i}^{2}-3M_{i}(m_{i-1}+m_{i})+3M_{i}^{2}].$$ (11)

The full Holladay integral is the inhomogeneous quadratic function of the variables *m_i_*, arrived at by adding the energies *E*(*f_i_*) of all partial functions *f_i_*(*x*). Introducing the vector of slopes

$$\tilde{m}={\left(m_{0},m_{1},\ldots ,m_{n}\right)}^{T},$$

we have in matrix notation

$$E(f)={\tilde{m}}^{T}H\tilde{m}-12{\tilde{M}}^{T}\tilde{m}+12c,$$

where

$$\begin{array}{l} H=\left|\begin{array}{llllll} \frac{4}{\Delta_{1}} & \frac{2}{\Delta_{1}} & & & & \\ \frac{2}{\Delta_{1}} & \frac{2}{\Delta_{1}}+\frac{2}{\Delta_{2}} & \frac{2}{\Delta_{2}} & & & \\ & \frac{2}{\Delta_{2}} & \frac{2}{\Delta_{2}}+\frac{2}{\Delta_{3}} & \frac{2}{\Delta_{3}} & & \\ & & \vdots & \vdots & \vdots & \\ & & & \frac{2}{\Delta_{n-1}} & \frac{2}{\Delta_{n-1}}+\frac{2}{\Delta_{n}} & \frac{2}{\Delta_{n}}\\ & & & & \frac{2}{\Delta_{n}} & \frac{4}{\Delta_{n}} \end{array}\right|,\\ \tilde{M}=\left|\begin{array}{lll} \frac{M_{1}}{\Delta_{1}} & & \\ \frac{M_{2}}{\Delta_{2}} & + & \frac{M_{1}}{\Delta_{1}}\\ \frac{M_{3}}{\Delta_{3}} & + & \frac{M_{2}}{\Delta_{2}}\\ & + & \vdots \\ \frac{M_{n}}{\Delta_{n}} & + & \frac{M_{n-1}}{\Delta_{n-1}}\\ & & \frac{M_{n}}{\Delta_{n}} \end{array}\right| \end{array}$$

and

$$c={\left(\frac{M_{1}}{\Delta_{1}}\right)}^{2}+{\left(\frac{M_{2}}{\Delta_{2}}\right)}^{2}+,\ldots ,+{\left(\frac{M_{n}}{\Delta_{n}}\right)}^{2}.$$

Minimizing this expression for the Holladay energy integral is to solve the linear system of equations

$$2H\tilde{m}=M$$ (12)

or

$$\begin{array}{c} \frac{2}{\Delta_{1}}m_{0}+\frac{1}{\Delta_{1}}m_{1}=\frac{3}{\Delta_{1}}M_{1}\\ \frac{1}{\Delta_{1}}m_{0}+\left(\frac{2}{\Delta_{1}}+\frac{2}{\Delta_{2}}\right)m_{1}+\frac{1}{\Delta_{2}}m_{2}=\frac{3}{\Delta_{1}}M_{1}+\frac{3}{\Delta_{2}}M_{2}\\ \frac{1}{\Delta_{2}}m_{1}+\left(\frac{2}{\Delta_{2}}+\frac{2}{\Delta_{3}}\right)m_{2}+\frac{1}{\Delta_{3}}m_{3}=\frac{3}{\Delta_{2}}M_{2}+\frac{3}{\Delta_{3}}M_{3}\\ \cdots =\cdots \\ \frac{1}{\Delta_{n}}m_{n-1}+\frac{2}{\Delta_{n}}m_{n}=\frac{3}{\Delta_{n}}M_{n} \end{array}$$

The first equation, in fact, may be restated by (10) as

$$\frac{1}{\Delta_{1}}U_{1}=\frac{1}{2}f_{1}^{\prime \prime }(x_{0})=0,$$

that is, as the requirement (2) that second derivatives vanish at the first knot. The last equation reflects the corresponding requirement at the last knot. The second equation is equivalent to

$$\frac{1}{\Delta_{1}}V_{1}+\frac{1}{\Delta_{2}}U_{2}=\frac{1}{2}f_{1}^{\prime \prime }(x_{1})-\frac{1}{2}f_{2}^{\prime \prime }(x_{1})=0,$$

implying second order differentiability at the interior knot *x*_1_. The remaining equations similarly enforce compatibility of the one-sided second derivatives at the remaining interior knots. This confirms the result of Holladay.

The reader should note that the system (12) for classical splines differs from the linear system mostly offered in the current literature. There the second order differentiability of the classical splines is already assumed and the spline functions are formulated in terms of those second order derivatives, *n_i_* = *f*″(*x_i_*), *i* = 0, …, *n*. However for weighted classical splines, to be encountered in Sec. 2.4.1, second order differentiability no longer holds, and a weighted version of the linear system (12) needs to be considered.

#### 2.2.1 Expressing the Lavery Integral

As to the integral of the absolute value of the second derivative, it is readily available in the case that the derivative does not change sign inside the subinterval:

$$\begin{array}{r} e(f_{i})=\int_{x_{i-1}}^{x_{i}}\left|f_{i}^{\prime \prime }(x)\right|dx=\left|\int_{x_{i-1}}^{x_{i}}f_{i}^{\prime \prime }(x)dx\right|\\ =\left|f_{i}^{\prime }(x_{i})-f_{i}^{\prime }(x_{i-1})\right|=\left|m_{i}-m_{i-1}\right|. \end{array}$$

This includes the case in which the polynomial is of lesser degree than cubic, and thus has constant second derivatives. This case is signaled by the vanishing of the quantity *D_i_* introduced earlier in (8).

The function *f*″(*x*), however, is a linear function in *x* and, unless constant, changes sign at some location $\hat{x}$, which also marks the location of the inflection point of *f_i_*(*x*). Suppose this location falls into the interior of the subinterval:

$$x_{i-1}<\hat{x}<x_{i}.$$

Then the integral has to be calculated as the sum of two integrals of linear functions:

$$e(f_{i})=\left|\int_{x_{i-1}}^{\hat{x}}f_{i}^{\prime \prime }(x)dx\right|+\left|\int_{\hat{x}}^{x_{i}}f_{i}^{\prime \prime }(x)dx\right|.$$

The integrals between the absolute value bars are of opposite signs, so that the total integral can be written as the absolute value of a difference of integrals

$$e(f_{i})=\left|\int_{x_{i-1}}^{\hat{x}}f_{i}^{\prime \prime }(x)dx-\int_{\hat{x}}^{x_{i}}f_{i}^{\prime \prime }(x)dx\right|.$$

These integrals can be separately evaluated as differences of slopes. Let

$${\hat{m}}_{i}=f_{i}^{\prime }(\hat{x})$$

denote the inflection slope, then — in the case of an interior inflection —

$$e(f_{i})=|2\hat{m}-m_{i-1}-m_{i}|.$$ (13)

The quantities *U_i_*, *V_i_* depend linearly on the two slopes *m_i_*_−1_, *m_i_*. Conversely, those slopes can be expressed in terms of *U_i_*, *V_i_*.

$$m_{i-1}=M_{i}-\frac{2}{3}U_{i}+\frac{1}{3}V_{i},m_{i}=M_{i}+\frac{1}{3}U_{i-1}-\frac{2}{3}V_{i}.$$ (14)

The next step is to express the inflection slope in terms of *U_i_*, *V_i_*. To this end, we express the inflection argument by its barycentric weights:

$$\hat{x}={\hat{\lambda }}_{i}x_{i-1}+{\hat{\mu }}_{i}x_{i},$$ (15)

From the expression (10) for *f_i_*″(*x*) it follows that

$${\hat{\lambda }}_{i}=\frac{V_{i}}{D_{i}},{\hat{\mu }}_{i}=\frac{U_{i}}{D_{i}},$$

where the denominator *D_i_* = *U_i_* + *V_i_* = 6*M_i_* − 3*m_i_*_−1_ −3*m_i_* has already been encountered in (8) as a quantity that vanishes if and only if the polynomial in question is parabolic or linear. Thus *D_i_* = 0 leads back to the previous case of no sign changes by the second derivative.

The weights ${\hat{\lambda }}_{i}$, ${\hat{\mu }}_{i}$ may now be inserted into the expression (7) for the derivative $f_{i}^{\prime }(x)$. This gives

$${\hat{m}}_{i}=\frac{V_{i}}{D_{i}}m_{i-1}+\frac{U_{i}}{D_{i}}m_{i}+\frac{V_{i}U_{i}}{D_{i}}.$$

Substituting for *m_i_*_−1_, *m_i_* according to (14) yields

$${\hat{m}}_{i}=M_{i}+\frac{U_{i}^{2}+V_{i}^{2}-V_{i}U_{i}}{3D_{i}}$$

as well as

$$e(f_{i})=\frac{U_{i}^{2}+V_{i}^{2}}{3|D_{i}|}=\frac{{\hat{\mu }}_{i}^{2}+{\hat{\lambda }}_{i}^{2}}{3}|D_{i}|.$$ (16)

in view of (19). In terms of the slopes *m_i_*_−1_, *m_i_*:

$$e(f_{i})=\left|\frac{18M_{i}^{2}-18M_{i}(m_{i-1}+m_{i})+5m_{i-1}^{2}+8m_{i-1}m_{i}+5m_{i}^{2}}{6M_{i}-3m_{i-1}-3m_{i}}\right|.$$

The above expressions for *e*(*f_i_*) are also valid if the inflections occur at the ends *x_i_*_−1_, *x_i_* of the interval of definition, reducing to *e*(*f_i_*) = |*m_i_* − *m_i_*_−1_|, in accordance with earlier results.

We are now ready to examine the full Lavery integral. At first blush, all that remains to be done is to sum over the partial integrals *e*(*f_i_*) in their various forms. We will show, however, that many terms of the expressions (13) cancel each other out as these partial integrals are added together. To this end, we distinguish five separate kinds of polynomials *f_i_*(*x*) depending on their behavior in the interior of the interval between its knots, *x_i_*_−1_ < *x* < *x_i_*:

- “Linear”; here $f_{i}^{\prime \prime }(x)=0$ throughout and *e*(*f_i_*) = 0
- “Convex”; here $f_{i}^{\prime \prime }(x)>0$ in the interior of the interval [*x_i_*_−1_, *x_i_*] and *e*(*f_i_*) = *m_i_* − *m_i_*_−1_
- “Concave”; here $f_{i}^{\prime \prime }(x)<0$ in the interior of the interval [*x_i_*_−1_, *x_i_*] and *e*(*f_i_*) = *m_i_*_−1_ − *m_i_*
- “Convex-concave”; here $f_{i}^{\prime \prime }(x)>0$ for $x<\hat{x}$, $f_{i}^{\prime \prime }(x)<0$ for $x>\hat{x}$ and $e(f_{i})=+\hat{m}-m_{i-1}-m_{i}$, also *D_i_* > 0
- “Concave-convex”; here $f_{i}^{\prime \prime }(x)<0$ for $x<\hat{x}$, $f_{i}^{\prime \prime }(x)>0$ for $x>\hat{x}$ and $e(f_{i})=-\hat{m}+m_{i-1}+m_{i}$, also *D_i_* < 0

The last two categories are the ones with an interior inflection point $\hat{x}$ and inflection slope $\hat{m}$.

The interior inflection points considered so far may not be the only inflection points of the spline function *f* (*x*). Inflections occur also at knots *x_i_*, 0 < *i* < *n* if a concave or convex-concave polynomial is followed by a convex or convex-concave polynomial and, analogously, if a convex or concave-convex polynomial is followed by a concave or concave-convex polynomial. We refer to such knots as “inflection knots”. To make matters more complicated, however, inflection may occur along an entire stretch of consecutive linear polynomials of equal slope, the inflection slope in this case, provided there are adjacent nonlinear polynomials at both ends of such a stretch exhibiting the same convexity/concavity pattern that would cause an inflection at a knot. In this case, we choose an arbitrary knot, say, the leftmost one in the linear stretch, as the inflection knot representing the inflection.

**Fig. 9:**
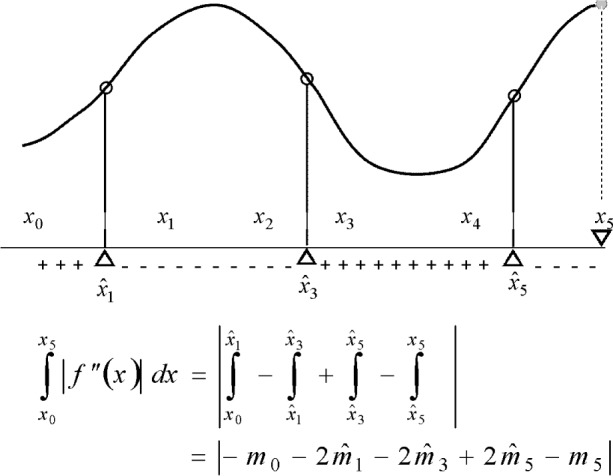
Lavery integral as expressed by inflection slopes and end slopes.

Clearly, slopes at interior knots that are not inflections cancel out as the expressions (13) for the Lavery integrals *e*(*f_i_*) for the partial spline functions *f_i_*(*x*) are added up. See Fig. 9 for an example. This leads to

**Observation A**: *The Lavery integral of a cubic spline function is the absolute value of an alternating sum of the inflection slopes and of the end slopes. Let*

$${\hat{m}}_{1},{\hat{m}}_{2},{\hat{m}}_{3},\ldots ,{\hat{m}}_{L},$$

*be the sequence of all inflection points identified above, sorted from left to right by their location. Then*

$$e(f)=\left|m_{0}+2\sum_{l=1}^{L}{\left(-1\right)}^{l}{\hat{m}}_{l}-{\left(-1\right)}^{L}m_{n}\right|.$$

(Note that the indices *l* of ${\hat{m}}_{l}$ do not refer to the interval in which they are located).

### 2.3 Properties of Lavery Splines

In this section, we gather some information about Lavery Splines, namely, interpolating spline functions in the affine space *S* which minimize their respective Lavery integrals. A first general observation concerns the convexity of the Lavery integral.

The Holladay and Lavery integrals (3) and (4) of a piecewise cubic spline function,

$$f(x)=f(m_{0},m_{1},\ldots ,m_{n};x)$$

are functions of the slope specifications *m_i_*:

$$E(f)=E(m_{1},m_{2},\ldots ,m_{n}),e(f)=e(m_{1},m_{2},\ldots ,m_{n}).$$

The quadratic function *E* (*f*) representing the Holladay integral can be shown to be positive definite and, therefore, strictly convex. Its minimum is unique on the affine manifold *S* interpolating spline functions. The restriction to *S* is, of course, necessary as the value of *E* (*f*) would not change if the spline function *f* (*x*) were modified by adding a linear function in *x*. Adding a non-zero linear function to the function *f* (*x*) would not preserve its interpolation property.

#### 2.3.1 Convexity

Next, we establish the convexity of the Lavery integral. It may be viewed as the extension of the *L*_1_ vector norm to a norm on the linear space *F*″ of second derivatives of spline functions:

$$e(f)={\left\|f^{\prime \prime }\right\|}_{1}.$$

The following generic seminorm properties are easily verified for the piecewise linear functions in *F*″,

$$\begin{array}{r} {\left\|f^{\prime \prime }\right\|}_{1}=0<=>f^{\prime \prime }=0\\ \left|\alpha {\left\|f^{\prime \prime }\right\|}_{1}\right|=\left|\alpha \right|{\left\|f^{\prime \prime }\right\|}_{1} \end{array}$$

along with the triangle inequality,

$${\left\|f^{(!)\prime \prime }+f^{(2)\prime \prime }\right\|}_{1}\leq {\left\|f^{(1)\prime \prime }\right\|}_{1}+{\left\|f^{(2)\prime \prime }\right\|}_{1}.$$

Suppose the two spline functions *f*^(1)^, *f*^(2)^ are actually two interpolating spline functions and, therefore, in the affine space *S*. Then their mean is again in *S* and, from the triangle inequality,

$$\left\|\frac{f^{(1)\prime \prime }+f^{(2)\prime \prime }}{2}\right\|\leq \frac{\left\|f^{(1)\prime \prime }\right\|+\left\|f^{(2)\prime \prime }\right\|}{2}$$

In terms of Lavery integrals,

$$\begin{array}{l} e\left(\frac{m_{0}^{\left(1\right)}+m_{0}^{\left(2\right)}}{2},\ldots ,\frac{m_{n}^{\left(1\right)}+m_{n}^{\left(2\right)}}{2}\right)\\ \leq \frac{e(m_{1}^{\left(1\right)},\ldots ,m_{n}^{\left(1\right)})+e(m_{1}^{1(1)},\ldots ,m_{n}^{\left(2\right)})}{2}, \end{array}$$

which establishes convexity. This leads immediately to

**Observation B**: *Any positive linear combination of Lavery splines for the same interpolation problem, — in particular, their mean —, is again a Lavery spline for this problem.*

#### 2.3.2 Uniqueness of Inflections

Contrary to the Holladay functional, which is strictly convex, the Lavery integral is not. As a result, uniqueness does not follow and, in fact, does not hold, as an example in Sec. 2.3.4 will show. Such minima of a convex function, however, must form a convex set.

Note that, in general,

$$\int \frac{\left|f^{(1)\prime \prime }(x)\right|+\left|f^{(2)\prime \prime }(x)\right|}{2}dx\geq \int \frac{\left|f^{(1)\prime \prime }(x)+f^{(2)\prime \prime }(x)\right|}{2}dx.$$

If both *f*^(1)^ and *f*^(2)^ are Lavery splines for the same interpolation problem, then so is their mean, and all three functions

$$f^{(1)\prime \prime },f^{\left(2\right)},\frac{f^{(1)\prime \prime }+f^{(2)\prime \prime }}{2}$$

return the same optimal value for their Lavery integrals. Thus equality holds in the above relation. This implies that both *f*^1^″(*x*) and *f*^2^″(*x*) have the same sign pattern:

$$f^{(1)\prime \prime }(x)\geq 0\;\text{if and only if}\;f^{(2)\prime \prime }(x)\geq 0.$$

This can be rephrased as

**Observation C**: *Two Lavery splines for the same optimization problem share essentially the same inflections: if one of them has an inflection point at* $x=\hat{x}$, *then so has the other unless it is linear at this point.*

#### 2.3.3 Free Ends of Lavery Splines

Here we will examine the free ends of Lavery splines, in particular, the cubic polynomial *f*_1_(*x*) and the corresponding first summand *e*(*f*_1_) of the Lavery integral *e*(*f*). As the end slope *m*_0_ may vary freely, it must optimize *e*(*f*_1_) while keeping the slope *m*_1_ fixed. This fact determines the behavior of Lavery splines at free ends. As seen in the previous section, the first summand *e*(*f*_1_) is a convex function in the variables *m*_0_, *m*_1_. If the slope *m*_1_ is held fixed, *e*(*f*_1_)(*m*_0_) is convex as a function in *m*_0_ alone. If the fixed slope equals the straight-line slope, then *m*_0_ = *m*_1_ = *M*_1_ obviously represents the optimal value for *m*_0_, since *f*_1_(*x*) in that case is a straight line with *e*(*f*_1_) = 0. We suppose, therefore, that *m*_1_ ≠ *M*_1_, and we examine the case, that *f*_1_(*x*) has an inflection $\hat{x}$ in the interval between the first two knots, $x_{0}\leq \hat{x}\leq x_{1}$.

Consider the function

$$\tilde{e}(f_{1})(m_{0})=\frac{U_{1}^{2}+V_{1}^{2}}{D_{1}},$$

where $\hat{m}$ as well as *U*_1_, *V*_1_ also depend on the variable *m*_0_. Clearly, the absolute value of $\tilde{e}(f_{1})$ is given by *e*(*f*_1_). Note that

$$\begin{array}{l} \frac{\partial \hat{e}(f_{1})}{\partial m_{0}}=\frac{V_{1}^{2}-U_{1}^{2}-6V_{1}U_{1}}{D_{1}^{2}}={\hat{\lambda }}_{1}^{2}-{\hat{\mu }}_{1}^{2}-6{\hat{\lambda }}_{1}{\hat{\mu }}_{1}\\ \;=6{\hat{\lambda }}_{1}^{2}-4{\hat{\lambda }}_{1}-1, \end{array}$$

in view of (10), and

$$\frac{\partial U_{1}}{\partial m_{0}}=-2,\frac{\partial V_{1}}{\partial m_{0}}=-1\frac{\partial D_{1}}{\partial m_{0}}=-3.$$

Solving the quadratic equation for $\hat{\lambda }$, and taking into account that $0\leq \hat{\lambda }\leq 1$, yields

$$\begin{array}{l} {\hat{\lambda }}_{1}=\frac{2+\sqrt{10}}{6}=0.860378,\\ {\hat{\mu }}_{1}=\frac{4-\sqrt{10}}{6}=0.139622. \end{array}$$ (17)

The value of *m*_0_ for which this value for ${\hat{\lambda }}_{1}$ is realized can be inferred from the general definition (15) of an inflection, giving the barycentric coordinate ${\hat{\lambda }}_{1}$ in terms of the slopes *m*_0_, *m*_1_, as follows:

$${\hat{\lambda }}_{1}(6M_{1}-3m_{0}-3m_{1})=3M_{1}-m_{0}-2m_{1},$$

which — for the particular value (17) of ${\hat{\lambda }}_{1}$ — yields the corresponding end-slope

$$m_{0}=\frac{10-\sqrt{10}}{5}M_{1}-\frac{5-\sqrt{10}}{5}m_{1}.$$ (18)

This value for *m*_0_ represents a locally unique stationary value of $\tilde{e}(f_{1})(m_{0})$.

Now $\tilde{e}(f_{1})(m_{0})=0$ would imply *e*(*f*_1_)(*m*_0_) = 0, and consequently linearity, that is, *m*_0_ = *m*_1_ = *M*_1_, which has been ruled out. By continuity, $\tilde{e}(f_{1})(m_{0})$ is either always positive or always negative, — in other words, either

$$e(f_{1})(m_{0})=+\tilde{e}(f_{1})(m_{0})$$

or

$$e(f_{1})(m_{0})=-\tilde{e}(f_{1})(m_{0}).$$

This implies that the value (18) for *m*_0_ is also a locally unique stationary value of *e*(*f*_1_)(*m*_0_). In view of the convexity of this function, it is also its minimizer. At the last end we differentiate

$$\hat{e}(f_{n})=\frac{U_{n}^{2}+V_{n}^{2}}{3D_{n}}$$ (19)

and find

$$\frac{\partial \hat{e}(f_{n})}{\partial m_{n}}=\frac{U_{n}^{2}-V_{n}^{2}-6U_{n}V_{n}}{D_{n}^{2}}=6{\hat{\mu }}_{n}^{2}-4{\hat{\mu }}_{n}-1.$$

Symmetrically, we thus have

$${\hat{\mu }}_{n}={\hat{\lambda }}_{1},{\hat{\lambda }}_{n}={\hat{\mu }}_{1}.$$

The equation

$${\hat{\mu }}_{n}(6M_{n}-3m_{n-1}-3m_{n})=3M_{n}-2m_{n-1}-m_{n}$$

thus yields a symmetric relationship to (18):

$$m_{n}=\frac{10-\sqrt{10}}{5}M_{n}-\frac{5-\sqrt{10}}{5}m_{n-1}.$$ (20)

This establishes

**Observation D**: *The free ends of Lavery splines are either linear functions or they contain an inflection in the interior of their interval of definition. The locations* ${\hat{x}}_{1}$, ${\hat{x}}_{n}$ *of these inflections are universally given, respectively, by*

$$\begin{array}{l} {\hat{x}}_{0}=\frac{2+\sqrt{10}}{6}x_{0}+\frac{4-\sqrt{10}}{6}x_{1},\\ {\hat{x}}_{n}=\frac{4-\sqrt{10}}{6}x_{n-1}+\frac{2+\sqrt{10}}{6}x_{n}. \end{array}$$

Observation D enables us to determine universal values for partial Lavery integrals at the end-intervals of Lavery splines. By (15), (16), (18), and again by (20), which implies

$${\hat{\mu }}_{1}^{2}+{\hat{\lambda }}_{1}^{2}={\hat{\lambda }}_{n}^{2}+{\hat{\mu }}_{n}^{2}=\frac{10-\sqrt{10}}{9},$$

we find

$$e(f_{1})=\frac{10-\sqrt{10}}{27}|D_{1}|,e(f_{n})=\frac{10-\sqrt{10}}{27}|D_{n}|.$$

Substituting for *m*_0_ and *m_n_*, respectively in *D*_1_ = 6*M*_1_ − 3*m*_0_ − 3*m*_1_ and *D_n_* = 6*M_n_* − 3*m_n_*_−1_ − 3*m_n_*,

$$D_{1}=\frac{3\sqrt{10}}{5}(M_{1}-m_{1}),D_{n}=\frac{3\sqrt{10}}{5}(M_{n}-m_{n-1}),$$

giving rise to

**Observation E**: *A necessary, but far from sufficient, condition for a spline function f to be a Lavery spline is that*

$$\begin{array}{l} e(f_{1})=\frac{2}{9}(\sqrt{10}-1)|M_{1}-m_{1}|,\\ e(f_{n})=\frac{2}{9}(\sqrt{10}-1)|M_{1}-m_{n-1}|. \end{array}$$

#### 2.3.4 Examples of Non-unique Lavery Splines

**Fig. 10:**
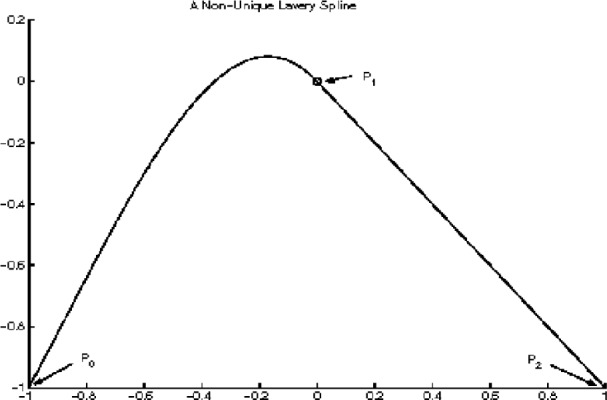
One of three Lavery splines for the same interpolation problem.

**Fig. 11:**
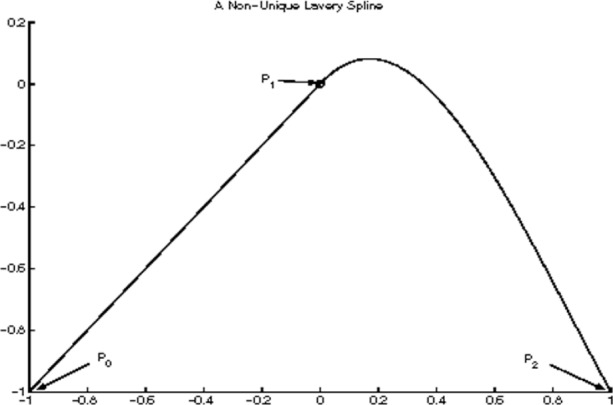
Second of three Lavery splines for the same interpolation problem.

**Fig. 12:**
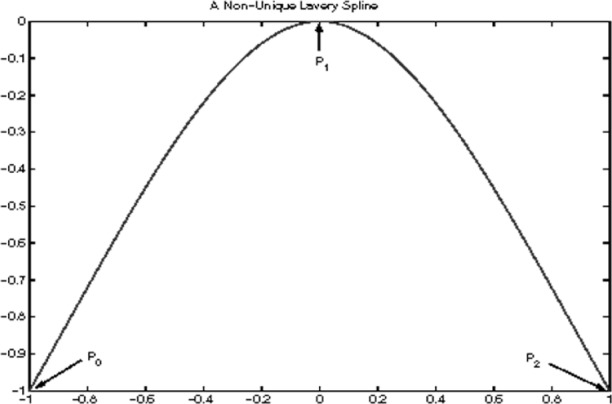
Third of three Lavery splines for the same interpolation problem.

In this section, we present an example in which the Lavery splines are not unique. Consider the three points (Figs. 10, 11, 12):

$$\begin{array}{l} P_{0}=(x_{0},y_{0})=(-1,-1)\\ P_{1}=(x_{1},y_{1})=(0,0)\\ P_{2}=(x_{2},y_{2})=(+1,-1). \end{array}$$ (21)

The associated interpolating cubic spline functions *f*(*x*) are then defined by their slopes

$$m_{0},m_{1},m_{2}$$

at these points. There are two subintervals with cubic polynomials

$$f_{1}(x),f_{2}(x),$$

each of them a free end. This determines the coordinates ${\hat{x}}_{1}$, ${\hat{x}}_{2}$ at inflections:

$${\hat{x}}_{1}=-\frac{2+\sqrt{10}}{6},{\hat{x}}_{n}=+\frac{2+\sqrt{10}}{6}.$$

Note that both partial Lavery integrals are end-integrals. For an interpolating spline function *f* (*x*) to be a Lavery spline it will be necessary by Observation E that

$$\begin{array}{l} e(f_{1})=\frac{2}{9}(\sqrt{10}-1)|1-m_{1}|,\\ e(f_{2})=\frac{\sqrt{10}-1}{9}|-1-m_{1}| \end{array}$$

so that

$$e(f)=\frac{2}{9}(\sqrt{10}-1)(|1-m_{1}|+|-1-m_{1}|).$$

Clearly

$$-1\leq m_{1}\leq 1$$ (22)

implies

$$|1-m_{1}|+|-1-m_{1}|=|(1-m_{1})+(-1-m_{1})|=2|m_{1}|\leq 2,$$

whereas either *m*_1_ < −1 or *m*_1_ > 1 would imply

$$|1-m_{1}|+|-1-m_{1}|=2\geq 2|m_{1}|.$$

Thus condition (22) characterizes all Lavery splines for the example.

Consider now any slope *m*_1_ from −1 through +1. For *m*_1_ = −1, we have by (18), (20), and in view of *M*_1_ = 1, *M*_2_ = −1:

$$m_{0}=\frac{+15-2\sqrt{10}}{5},m_{2}=-1.$$

Symmetrically, we find for *m*_1_ = +1 that

$$m_{0}=+1,m_{2}=\frac{-15+2\sqrt{10}}{5}.$$

These slopes, respectively, determine the two extreme Lavery splines, because each partial function is at a free end, and is optimized according to Observation D in the previous section. The two resulting Lavery splines are extreme in that each has a straight line segment as a partial function.

Using Hermite’s formula (6) and substituting for *x*, we find for the choice *m*_1_ = −1,

$$\begin{array}{l} f_{1}(x)=-\frac{2\sqrt{10}}{5}x^{3}-\frac{10+2\sqrt{10}}{5}x^{2}-x\\ f_{2}(x)=-x. \end{array}$$

For *m*_1_ = +1, the resulting Lavery spline is the symmetric image of the previous one:

$$\begin{array}{l} f_{1}(x)=+x\\ f_{2}(x)=-\frac{2\sqrt{10}}{5}x^{3}-\frac{10+2\sqrt{10}}{5}x^{2}+x. \end{array}$$

Both splines are shown in Figs. 10, 11. The self-symmetric spline from the choice *m*_1_ = 0 is shown in Fig. 12. In the latter case,

$$m_{0}=\frac{10-\sqrt{10}}{5}=-m_{2},$$

and

$$\begin{array}{l} f_{1}(x)=-\frac{\sqrt{10}}{5}x^{3}-\frac{5+\sqrt{10}}{5}x^{2}\\ f_{2}(x)=+\frac{\sqrt{10}}{5}x^{3}-\frac{5+\sqrt{10}}{5}x^{2}. \end{array}$$

This Lavery spline is the mean of the two splines with linear free ends. This is an instance of observation B about positive linear combinations of Lavery splines in Sec. 2.3.1.

### 2.4 Computing Univariate Lavery Splines

We now turn our attention to the computation of Lavery splines. The commonly used approach (see Lavery [11,12]) is to minimize the Lavery integral in discretized form, say,

$$\int_{x_{0}}^{x_{n}}|f^{\prime \prime }(x)|dx=\sum_{i}\frac{\Delta_{i}}{k_{i}}\sum_{k=1}^{k_{i}}\left|f^{\prime \prime }(x_{i-1}+\frac{k-1}{k_{i}}\Delta_{i})\right|,$$ (23)

where the integrand is sampled in each subinterval [*x_i_*_−1_, *x_i_*] at *k_i_* equidistant points. Now

$$\begin{array}{r} x_{i-1}+\frac{k-1}{k_{i}}\Delta_{i}=\frac{(k_{i}-k+1)x_{i-1}+(k+1)x_{i}}{k_{i}}\\ =\lambda_{i,k}x_{i-1}+\mu_{i,k}x_{i}\lambda_{i,k}+\mu_{i,k}=1. \end{array}$$

Thus

$$\begin{array}{l} \int_{x_{0}}^{x_{n}}f^{\prime \prime }(x)dx=\sum_{i}\frac{\Delta_{i}}{k_{i}}\sum_{k-1}^{k_{i}}|\lambda_{i,k}U_{i}+\mu_{i,k}V_{i}|\\ =\sum_{i}\frac{\Delta_{i}}{k_{i}}\sum_{k-1}^{k_{i}}\left|\lambda_{i,k}(3M_{i}-2m_{i-1}-m_{i})+\mu_{i,k}(3M_{i}-m_{i-1}-2m_{i})\right| \end{array}$$

The Lavery integral is discretized as a sum of absolute values of the variables *m_i_* of the minimization. Minimizing such an expression is a Linear Programming problem. Many satisfactory methods for solving it are available, such as the Simplex Method or Interior Point Methods. The accuracy of the discretization increases with the number of sample points, but computational effort increases accordingly.

For that reason, and also to motivate an analogous approach in the bivariate case, we are proposing to minimize a different approximation to the Lavery integral, one that takes advantage of the ease of computation offered by energy minimization.

By the mean value theorem of integral calculus, there exist arguments *u_i_* such that

$$\int_{x_{i-1}}^{x_{i}}f_{i}^{\prime \prime }{\left(x\right)}^{2}dx=\Delta_{i}(f_{i}^{\prime \prime }{\left(u_{i}\right)}^{2},i=1,\ldots ,n.$$

we then propose to approximate the Lavery integral by the following Riemann sum:

$$\int_{x_{0}}^{x_{n}}|f^{\prime \prime }(x)|dx\approx \sum_{i}\Delta_{i}|f_{i}^{\prime \prime }(u_{i})|.$$ (24)

In contrast to the approximation (23) by discretization, this approximation does not offer the option of further refinement, unless the interpolation problem itself is changed by adding additional knots and ordinates. In a sense, it approximates the Lavery paradigm itself rather than the Lavery integral. We still use, however, the term “approximate Lavery integral” for our proposed approximation (24).

#### 2.4.1 An Iterative Algorithm for Approximate Lavery Splines

For the purpose of computation, we rewrite the nonzero terms in (46)

$$\begin{array}{l} \Delta_{i}\sqrt{f_{i}^{\prime \prime }{\left(u_{i}\right)}^{2}}=\sqrt{\Delta_{i}}\frac{\Delta_{i}f_{i}^{\prime \prime }{\left(u_{i}\right)}^{2}}{\sqrt{\Delta_{i}f_{i}^{\prime \prime }{\left(u_{i}\right)}^{2}}}\\ \;=\frac{\sqrt{\Delta_{i}}}{\sqrt{\int_{x_{i-1}}^{x_{i}}f_{i}^{\prime \prime }{\left(x\right)}^{2}dx}}\int_{x_{i-1}}^{x_{i}}f_{i}^{\prime \prime }{\left(x\right)}^{2}dx. \end{array}$$

This expression suggests an iterative approach. Starting with the classic spline *f*^(0)^(*x*), a sequence of interpolating spline functions is generated in hopes to converge towards the approximate Lavery integral (24)

$$f^{\left(0\right)}(x),f^{\left(1\right)}(x),\ldots ,f^{\left(1\right)}(x),\ldots$$

with associated partial Holladay integrals

$$E^{f_{i}^{\left(l\right)}}={\int_{x_{i-1}}^{x_{i}}\left(f_{i}^{(l)\prime \prime }(x)\right)}^{2}dx,i=1,\ldots ,n.$$

At each step *l* = 0, 1, 2, …, weights

$$w_{i}^{\left(l\right)}=\sqrt{\frac{\Delta_{i}}{E(f_{i}^{\left(l\right)})},}i=1,\ldots ,n,$$

are introduced. Given the function *f*^(^*^l^*^)^, the subsequent function *f*^(^*^l^*^+1)^ is determined as the solution to the following minimization problem:

$$\min\sum_{i}w_{i}^{\left(l\right)}\int_{x_{i-1}}^{x_{i}}{\left(f_{i}^{(l+1)\prime \prime }(x)\right)}^{2}dx.$$ (25)

This approach raises the question what to do if $E(f_{i}^{\left(l\right)})$ vanishes as the corresponding weight is then not defined?

Simply ignoring such terms may prematurely lock in straight line segments between knots. What first comes to mind is to specify a limit *ε* > 0 and boost lower values of $E(f_{i}^{\left(l\right)})$ to this level. A more diligent procedure might be to start with all weights at value 1, — the weight setting that yields the initial classical spline according to Holliday’s observation —, and then progressively increase the use of weights. Such strategies remain to be explored.

In general, adding up partial Holliday integrals, each with weight, say, *w_i_* leads again to an expression of the energy of a physical structure: a collection of thin beams of different thicknesses given by *w_i_*, respectively, and welded together at knots. Minimizing this energy expression requires an adjustment to the linear system (12).

The weighted energy of the partial spline functions *f_i_*(*x*) is just the product of the straight Holliday integral and the respective weight:

$$E_{W}(f_{i})=w_{i}E(f_{i}).$$

The total weighted energy is thus given by

$$E_{W}(f)=\sum_{i}E_{w}(f_{i})=\sum_{i}w_{i}E(f_{i}).$$

This means that in the expression (11) for *E*(*f_i_*), the factor

$$\frac{4}{\Delta_{i}}$$

is affected as it is multiplied by *w_i_*. In other words, 1the substitution,

$$\frac{1}{\Delta_{i}}\to \frac{w_{i}}{\Delta_{i}},$$

transforms the linear system (12) for minimizing *E*(*f*) into the linear system for minimizing *E_W_*(*f*):

$$\begin{array}{c} \frac{2w_{1}}{\Delta_{1}}m_{0}+\frac{w_{1}}{\Delta_{1}}m_{1}=\frac{3w_{1}}{\Delta_{1}}M_{1}\\ \frac{w_{1}}{\Delta_{1}}m_{0}+\left(\frac{2w_{1}}{\Delta_{1}}+\frac{2w_{2}}{\Delta_{2}}\right)m_{1}+\frac{w_{2}}{\Delta_{2}}m_{2}=\frac{3w_{1}}{\Delta_{1}}M_{1}+\frac{3w_{2}}{\Delta_{2}}M_{2}\\ \frac{w_{2}}{\Delta_{2}}m_{1}+\left(\frac{2w_{2}}{\Delta_{2}}+\frac{2w_{3}}{\Delta_{3}}\right)m_{2}+\frac{w_{3}}{\Delta_{3}}m_{3}=\frac{3w_{2}}{\Delta_{2}}M_{2}+\frac{3w_{3}}{\Delta_{3}}M_{3}\\ \cdots =\cdots \\ \frac{w_{n}}{\Delta_{n}}m_{n-1}+\frac{2w_{n}}{\Delta_{n}}m_{n}=\frac{3w_{n}}{\Delta_{n}}M_{n} \end{array}$$ (26)

Note that the weighted classic splines are, in general, not twice differentiable at the knots. However, due to the fact that the first and the last equations have common factors *w*_o_, *w_n_*, respectively, they are equivalent to the corresponding equations in the Holladay system (12): free ends thus have zero second derivatives in the weighted case, too. Again, this is to be expected from Physics.

Note also that some commonly used methods for determining classic splines such as B-splines or linear systems formulated in terms of second derivatives at knots do not carry over to the weighted case. However, the above linear system is still “banded”, and many excellent methods are known for its solution. To solve this system we used the venerable Gauss-Seidel method, not just for ease of programming, but also because it seems to work for bivariate weighted splines. Its advantage lies in the fact that the matrix of the linear system need not be changed and can be read, so to speak, in sequence. This is important for the very large systems likely to arise in the bivariate case. In the univariate case, the convergence behavior is well understood (See Varga [22]). Using an iterative method for solving the class of linear systems above will result in a two-tiered iteration procedure: an “outer” iteration, developing new sets of weights, and an “inner” iteration, solving the resulting linear system. Such procedures can be “balanced”, that is, the inner iteration may be terminated at a lower accuracy level during the early stages of the outer iteration and may be carried to a higher level of accuracy as the outer method approaches convergence. This is an added advantage of an iterative method for solving the linear systems at hand.

Note finally, that the approximate Lavery method proposed here will definitely not converge to the optimal Lavery integral. This is because the approximate solutions are based on weighted classic splines, and therefore have vanishing second derivatives at the free ends. The second derivatives of Lavery splines, on the other hand, assume their inflections in interiors of the end interval (See Observation D).

**Observation F**: *Unless a free end function of an approximate Lavery spline is linear, it disagrees with the corresponding end function of the true Lavery spline in that the latter has an inflection in the interior of at least one end interval, whereas the former assumes its inflection at the end knot.*

The justification for introducing the approximate Lavery splines concept lies in its computational ease and the fact that it appears to retain the anti-oscillatory dynamic of the original Lavery concept. In fact, the examples in Figs. 5, 6, and 8 were calculated using the approximate algorithm outlined in this section.

## 3. Bivariate Spline Interpolation

The development of bivariate splines parallels that of univariate ones to a large extent. For both, the first step is to specify a linear space *F* of bivariate spline functions *f* (*x*, *y*), defined on a polygonally partitioned region

Ω

In view of our emphasis on the TIN framework of triangulations, however, we choose for our discussion a rectangular region Ω and an irregular set of “knots” in Ω,

$$v_{i}=(x_{i},y_{i})\in \Omega ,i=1,\ldots ,n.$$

An interpolation problem results, if “elevations” *z_i_* are specified at the vertices *v_i_* = (*x_i_*, *y_i_*).

The region Ω is then partitioned into triangles

$$t_{k}.$$

The vertices

$$\left(x_{i_{k,1}},y_{i_{k,1}}\right),\left(x_{i_{k,2}},y_{i_{k,2}}\right),\left(x_{i_{k,3}},y_{i_{k,3}}\right)$$

of any such triangle *t_k_* are among the specified knots, and those are the only knots in this triangle. In this fashion, a “triangulation” of the region Ω is obtained. Spline functions are then defined by installing partial functions

$$f_{x}(x,y),\;k=1,\ldots ,m$$

in their respective triangles *t_k_* of the triangulation.

**Fig. 13:**
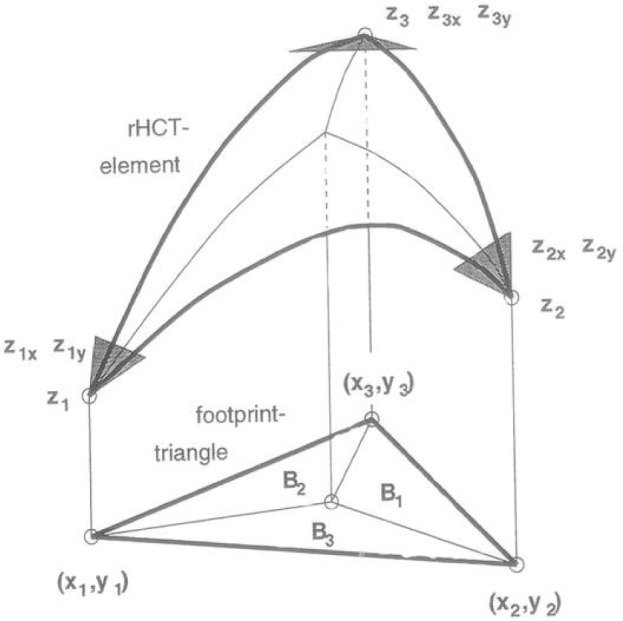
Reduced Hsieh-Clough-Tocher (rHCT) element. *zi_x_*, *zi_y_* denote the partial slopes *zx_i_*, *zy_i_* (27).

**Fig. 14:**
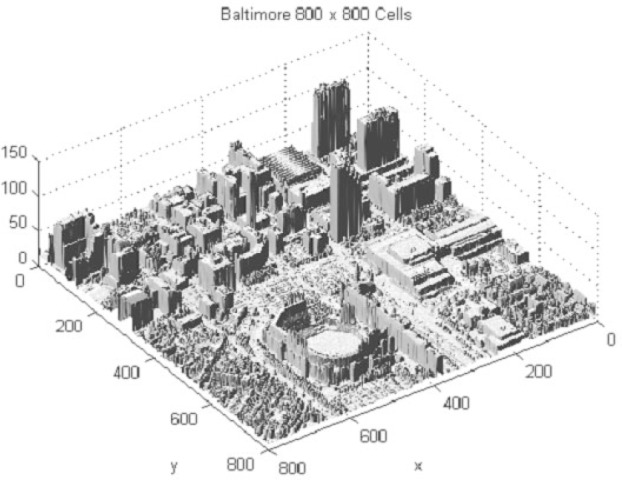
Urban scene (Baltimore): Gibbs phenomena are visible.

Following C. Lawson [14,15], we install above each triangle *t_k_* a reduced Hsieh-Clough-Tocher (rHTC) element. The reduced Hsieh-Clough-Tocher (rHCT) element, displayed in Fig. 14, provides a means for representing smooth TIN surfaces (see Lawson [14,15]). The rHCT element is defined as a function over a triangle in the *x*, *y*-plane, and is fully determined by the elevations and the partial slopes or derivatives at triangle corners. The triangle is divided into three subtriangles with their common vertex at the centroid of the three corners of the full triangle. In each of the three subtriangles the rHCT function is a bivariate cubic function. Together, these functions form a *C*^1^ function on the full triangle (Fig. 13). This smooth rHCT function is furthermore constrained by the linear perpendicularity condition on the derivatives orthogonal to the outside boundary edges of the triangular element: along each such edge, these derivatives are required to be linear functions. It follows that the orthogonal derivatives along an edge are fully determined by their values at the ends of the edges. These values are inherent to both triangles adjacent to the edge so that the orthogonal derivatives coincide when calculated within each of the two triangles independently. The above condition thus provides the key property ensuring smoothness of a rHTC surface. As will be seen in Sec. 3.1.2, the restriction inherent in the linear perpendicularity condition may be more severe than commonly expected.

To this end, “elevations”

$$z_{i}$$

and “partial slopes”

$$zx_{i},zy_{i},i=1,\ldots ,n,$$ (27)

are prescribed at the knots *v_i_*. As the vertices of each triangle *t_k_* are among the specified knots, the corresponding elevations

$$z_{i_{k,1}},z_{i_{k,2}},z_{i_{k,3}}$$

and partial slopes

$$xz_{i_{k,1}},xy_{i_{k,1}},zx_{i_{k,2}},zy_{i_{k,2}},zx_{i_{k,3}},zy_{i_{k,3}}.$$

furnish the parameters necessary for defining the rHTC element *f_k_*(*x*, *y*). As pointed out above in the current section, each specification of elevations and partial slopes at the knots yields a *C*^1^ function *f* (*x*, *y*), which also represents a smooth surface over the region of definition Ω. All such functions, — relating to the given triangulation of Ω —, constitute a linear space of “spline functions” and their respective derivative linear spaces

$$F,F_{x},F_{y},F_{xx},F_{xy},F_{yy}.$$

If a specific set of elevations *z_i_* is to be interpolated, then those spline functions which meet these elevations constitute an affine space of “interpolating spline functions” and its derivative affine spaces

$$S,S_{x},S_{y},S_{xx},S_{xy},S_{yy}.$$

Note, again, that second derivatives are not fully defined for rHTC elements. Indeed, along edges separating two subtriangles of an rHTC element as well as along edges separating two triangles of the triangulation there may be two different values in contention depending from which side a point on such an edge is approached. For purposes of integration, this is not an issue since the discrepancies are restricted to a set of measure zero, so that the integrals considered below are still well defined.

### 3.1 Paradigms for Non-Oscillatory Bivariate Splines

For the purpose of interpolation, the elevations *z_i_* at knots are specified, and the task at hand is to determine the partial slopes *zx_i_*, *zy_i_* so as to arrive at a “best” interpolating spline function. Such an optimal spline function is then referred to as a “(bivariate) spline”. Obviously, there are various kinds of splines depending on the choice of the linear space of spline functions and the choice of the optimization criterion.

Paralleling the classical univariate approach, “thin plate” minimization exemplifies the “classical” approach, and the resulting spline will be referred to as the “classical spline”, the functional requiring minimization being

$$\tilde{E}(f)=\int_{\Omega }(f_{xx}^{2}+2f_{xy}^{2}+f_{yy}^{2})dxdy.$$ (28)

An approach to computing the surface energy formula has been announced by McClain and Witzgall [16] for rHCT element. A newer version of the report is currently being prepared by McClain, Witzgall, and Gilsinn [17].

The authors believe that, contrary to univariate classical splines, bivariate classical splines as defined above are, in general, not in class *C*^2^, that is, twice differentiable. Nevertheless they are “stiff” and thus subject to the dreaded spurious oscillations and Gibbs phenomena. This can be seen along edges of buildings in the urban data set of Baltimore, MD, given in Fig. 14. Lavery and Gilsinn [13] were able to address this problem by extending his paradigm for univariate nonoscillatory splines to the bivariate case by minimizing the functional

$$e(f)=\int_{\Omega }(|f_{xx}|+2|f_{xy}|+|f_{yy}|)dxdy.$$ (29)

Again, the squares of second derivatives in an energy integral, — here $\tilde{E}(f)$ as defined in (28) —, are replaced by absolute values. We will refer to the so defined splines also a “(bivariate) Lavery splines”.

In view of the non-uniqueness result for univariate Lavery splines, the bivariate Lavery splines are expected not to be unique either, as will be discussed below. Therefore, the Lavery paradigm typically includes a regulatory term such as adding a small multiple of *f*′(*x*, *y*)^2^. The resulting bivariate Lavery splines have produced oscillation-free interpolations and approximations in a large array of applications. They are computed by discretizations leading to very large linear programming problems.

For large surface generation tasks based on interpolation, the resulting computational effort may be prohibitive. Moreover, the bivariate Lavery integral (29) functional is not invariant under rotation of the coordinate system. The authors have therefore proposed an alternate extension of the univariate Lavery paradigm to the bivariate case:

$$\tilde{e}(f)=\int_{\Omega }\sqrt{f_{xx}^{2}+2f_{xy}^{2}+f_{yy}^{2})}dxdy.$$ (30)

Note, that this paradigm is indeed an extension of the univariate Lavery paradigm. Indeed,

$$\int |f^{\prime \prime }(x)|dx=\int \sqrt{f^{\prime \prime }{\left(x\right)}^{2}}dx,$$

so that the integrand of the univariate Lavery integral may also be interpreted as the squareroot of an energy, — in this case, the elastic energy of a thin beam. Replacing that energy expression by that for a thin plate, yields the alternate extension.

#### 3.1.1 Properties of the Alternative Bivariate Paradigm

The differential expression

$$(f_{xx}^{2}+2f_{xy}^{2}+f_{yy}^{2})dxdy$$

is readily seen to be rotation invariant, as is to be expected, given its physical meaning as the integrand for the representation (28) of the thin plate energy. It follows that every differential expression of the form

$$\Phi (f_{xx}^{2}+2f_{xy}^{2}+f_{yy}^{2})dxdy$$

is also rotation invariant. This leads to the

**Observation G**: *The Alternate Bivariate Functional* $\tilde{e}(f)$ *is invariant under a rotation in the x*, *y*-*plane.*

Let *f* = *f* (*x*, *y*), *g* = *g*(*x*, *y*) ∈ *S* be two interpolating spline functions. Invoking the general vector inequality ‖*u*‖ + ‖*v*‖ ≥ ‖*u* + *v*‖, we find

$$\begin{array}{l} \frac{\sqrt{f_{xx}^{2}+2f_{xy}^{2}+f_{yy}^{2}}+\sqrt{g_{xx}^{2}+2g_{xy}^{2}+g_{yy}^{2}}}{2}\\ \geq \sqrt{{\left(\frac{f_{xx}+g_{xx}}{2}\right)}^{2}+2{\left(\frac{f_{xy}+g_{xy}}{2}\right)}^{2}+{\left(\frac{f_{yy}+g_{yy}}{2}\right)}^{2}}, \end{array}$$

so that

$$\begin{array}{l} \frac{1}{2}\int_{\Omega }\sqrt{f_{xx}^{2}+2f_{xy}^{2}+f_{yy}^{2}}dxdy+\frac{1}{2}\int_{\Omega }\sqrt{g_{xx}^{2}+2g_{xy}^{2}+g_{yy}^{2}}dxdy\\ \geq \int_{\Omega }\sqrt{{\left(\frac{f_{xx}+g_{xx}}{2}\right)}^{2}+2{\left(\frac{f_{xy}+g_{xy}}{2}\right)}^{2}+{\left(\frac{f_{yy}+g_{yy}}{2}\right)}^{2}}, \end{array}$$

and in terms of the spline functionals,

$$\frac{\tilde{e}(f)+\tilde{e}(g)}{2}\geq \tilde{e}\left(\frac{f+g}{2}\right).$$

Since *S* is an affine space, the mean (*f* + *g*)/2 is again in *S*. Based on the above inequality, we thus have

**Observation H**: *The alternate Bivariate functional (30) is a convex functional. In particular, as expressed in terms of the partial slopes*,

$$\tilde{e}(f)=\tilde{e}(zx_{1},zy_{1},\ldots ,zx_{n},zy_{n}),$$

*it is a convex function in terms of these partial slopes.*

#### 3.1.2 A Related Non-Uniqueness Result

We do not know at this point, whether the convexity is moreover strict. We expect that it is not. Strict convexity would imply uniqueness of minima and, again, we do not believe that this is the case. However, an example for such non-uniqueness is eluding us for our specific space of rHTC spline functions.

**Fig. 15:**
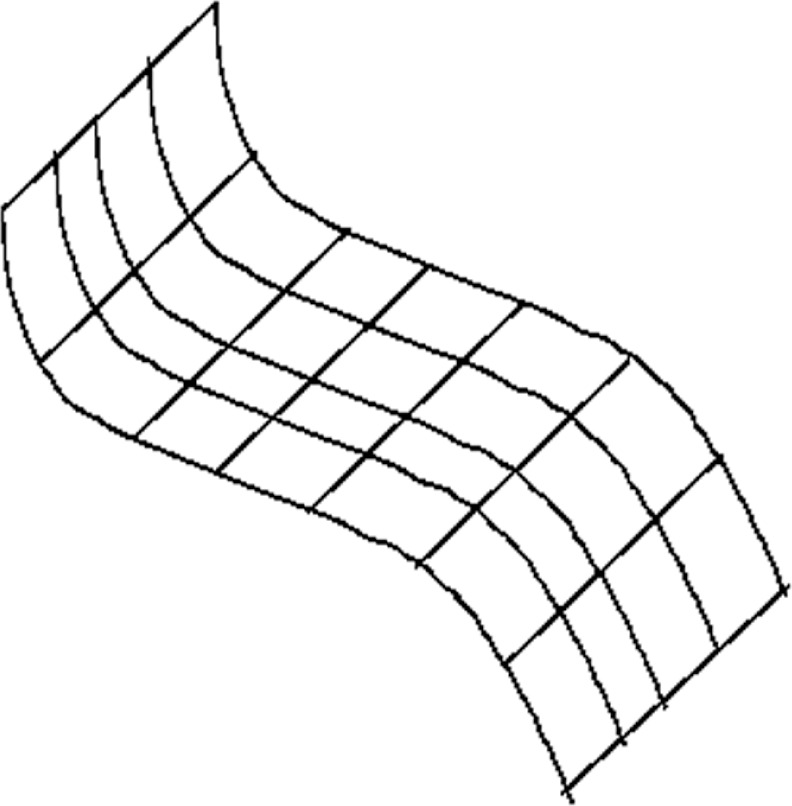
Non-uniqueness of a minimum for a bivariate function.

For different bivariate functions, however, it is possible to establish non-uniqueness of a minimum. Consider a function

s(x,y),−1≤x≤+1,0≤y1

representing a rectangular strip bent only in the direction of the *x*-axis. In the direction of the *y*-axis, the function *s*(*x*, *y*) is constant (Fig. 15), in particular, for all *y*,

s(−1,y)=−1,s(0,y)=0,s(+1,y)=−1.

The function *s*(*x*, *y*) thus interpolates the values −1, 0, −1 along the contour lines in the direction of the *y*-axis at the arguments *x* = −1, 0, +1. Given any Lavery spline *f* (*x*) considered in Sec. 2.3.3 as interpolating the example (21). Then it is readily seen, that any function

s(x,y)=f(x)

minimizes both bivariate functionals: *e*(*s*), $\tilde{e}(s)$. For functions corresponding to strips bent in only the strip direction, these bivariate functionals have thus multiple minima. This makes a strong case for non-uniqueness bivariate splines minimizing the functionals (29) and (30). However, we were not able to pinpoint non-uniqueness for the rHTC based spline functions considered in this section. In particular, we were surprised to realize that the function *s*(*x*, *y*) cannot be generated using rHTC elements. As this fact points to an important limitation of using rHTC elements, we formulate the

**Observation J**: *The surface function of a rectangular strip bent only in the strip direction cannot be represented by means of rHTC elements.*

### 3.2 Computing Bivariate Splines Based on an Alternate Paradigm

The proposed alternate functional (53) is still expensive to minimize. In analogy to the procedure described in Sec. 2.4.1, we propose to approximate that functional as follows. Let

$$\Delta_{k}$$

denote the area of the triangle *t_k_* of the triangulation. Then the integral of each partial spline function or element *f_k_* = *f_k_*(*x*, *y*) is individually approximated.

$$\begin{array}{l} \int_{t_{k}}\sqrt{f_{kxx}^{2}+2f_{kxy}^{2}+f_{kyy}^{2}}dxdy\\ =\frac{\sqrt{\Delta_{k}}}{\sqrt{\int_{t_{k}}(f_{kxx}^{2}+2f_{kxy}^{2}+f_{kyy}^{2}dxdy}}\int_{t_{k}}(f_{kxx}^{2}+2f_{kxy}^{2}+f_{kyy}^{2}dxdy. \end{array}$$

This approximation suggests an iterative procedure based on minimizing weighted energy expressions: Starting with the classic spline *f*^(0)^(*x*, *y*), a sequence of interpolating spline functions is generated in hopes to converge towards the alternate Lavery integral (30),

$$f^{\left(0\right)}(x,y),f^{\left(1\right)}(x,y),\ldots ,f^{\left(l\right)}(x,y),\ldots$$

with associated partial integrals

$$\tilde{E}(f_{k}^{\left(l\right)})=\int_{t_{k}}\left(f_{kxx}^{{\left(l\right)}^{2}}+2f_{kxy}^{{\left(l\right)}^{2}}+f_{kyy}^{{\left(l\right)}^{2}}\right)dxdy,k=1,\ldots ,m$$

At each step *l* = 0, 1, 2, …, weights

$$w_{k}^{\left(l\right)}=\sqrt{\frac{\Delta_{i}}{\tilde{E}(f_{k}^{\left(l\right)})}},k=1,\ldots ,m,$$

are introduced. Given the function *f*^(^*^l^*^−1)^, the subsequent function *f*^(^*^l^*^)^ is determined as the solution to the following minimization problem (compare (25)):

$$\min\sum_{i}w_{i}^{\left(l-1\right)}\int_{t_{k}}(f_{kxx}^{(l)2}+2f_{kxy}^{(l)2}+f_{kyy}^{(l)2})dxdy.$$ (31)

For the exploratory implementation reported here, the above minimization of a quadratic function in the partial slopes

$$zx_{i_{k,1}},zy_{i_{k,1}},zx_{i_{k,2}},zy_{i_{k,2}},zx_{i_{k,3}},zy_{i_{k,3}}.$$

is again formulated as solving a linear system of equations in these variables, and Gauss-Seidel iteration provided a workable option. Just as discussed in the univariate case, there are two iterative processes, one “on top of” of the other. The “inner iteration” aims to solve the linear system of equations in order to solve the intermediate minimization problem (31), while the “outer” iteration sequentially creates spline functions expected to converge toward a limit that approximates the alternate Lavery spline defined by the minimization of the alternate Lavery functional (30).

This approach raises the same issues as its parallel in the univariate case. The above iterative approximation based on consecutive minimization of weighted sums of integrals (31) cannot be expected to converge exactly to the alternate bivariate Lavery spline. The argument relies on Observation F pertaining to the univariate case. Each iterate *f*^(^*^l^*^)^(*x*, *y*) minimizes the energy of a physical structure consisting of thin plates of various thicknesses, namely the weights. Any minimizing shape of that structure, however, is known to have some vanishing second derivatives at its boundary. Univariate Lavery splines do not have this property, and we do not expect bivariate Lavery splines to have this property, either.

Provisions need to be made should one of the integrals $\tilde{E}(f_{k}^{\left(l-1\right)})$ vanish. This is a serious problem because very large or “infinite” weights will cause certain rHTC elements to remain stuck in linear form. In addition, limits of bivariate Lavery splines, in the original as well as the alternate formulation, may not be unique (see Sec. 3.1). These issues call for regularization procedures which are adequate for particular kinds of applications.

Indeed, spurious spikes were observed for the applications demonstrated here. The problem was solved by interspersing an averaging step, where the partial slopes were replaced by averages of neighboring slopes.

**Fig. 16:**
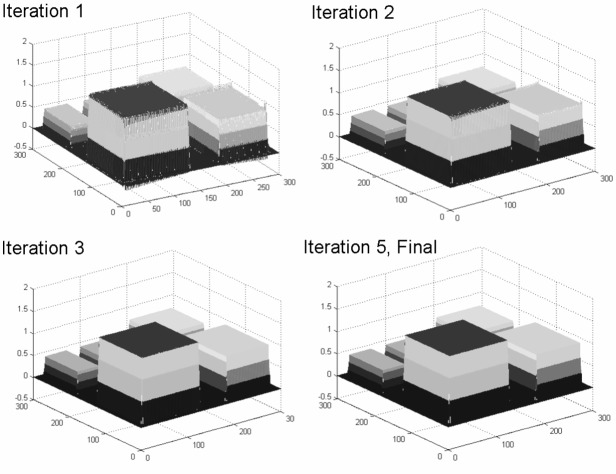
Example of 2-D Lavery splines.

While our experiences with approximating alternate bivariate Lavery splines (Fig. 16) were encouraging, in particular, for very large data sets, much work needs to be done.

The accuracy performance of the Gauss-Seidel iteration is a major issue. Simple examples show that initial steps are not even contracting. When does contraction start? Does it keep contracting after that? Much research has been aimed at these questions. These analyses of the convergence properties of the Gauss-Seidel iteration, however, provide estimates of the number of iterations required to solve linear equations to a specified accuracy which are much too large to be practical. To be practical, the number of “passes” through the entire system of linear equations on the order of 10 or 20, has to be sufficient.

In our experience, relatively few steps of the “outer” iteration were sufficient to provide qualitatively satisfactory results. However, we do not have formal proof of convergence.

Finally, and perhaps most importantly, a better understanding of the necessary regularization devices needs to be developed.

## 4. Conclusions

In this paper we have investigated some properties of non-oscillatory splines introduced by John Lavery [11,12]. These splines, called in this paper Lavery splines, minimize what we have termed the Lavery integral (4). We have seen that the minimizing spline for (4) does indeed model sharp edges and jumps in data without introducing the “Gibbs phenomenon” at the corners. We have shown that the Lavery integral and the associated Lavery splines satisfy a number of properties. First, we have shown that the Lavery integral of a cubic spline function is the absolute value of an alternating sum of inflection slopes and of the end slopes. Next, we showed that any positive linear combination of Lavery splines for the same interpolation problem is again a Lavery spline for the same problem. Furthermore, two Lavery splines for the same optimization problem share essentially the same inflection points and that the free ends of Lavery splines are either linear functions or they contain an inflection point in the interval of definition.

Two algorithms for estimating Lavery splines have also been considered. The first algorithm introduced by John Lavery [11,12] reduces to solving a least absolute value minimization problem for which he used an interior point method for linear programming to obtain the minimum spline coefficients. The absolute value minimization was based on a “discretization” which leads to a very large number of variables as sufficiently smaller discretizations were considered. The extension of this method to bivariate Lavery splines also led to computationally intensive compute times even for moderate data sizes. The authors have introduced a modified approach based on an iterated weighted least squares algorithm. Although the minimizing spline this algorithm produces is not a Lavery spline it is an approximation that also produces sharp edges without the “Gibbs phenomenon.”

In the bivariate case the Lavery integral minimizes the integral of the sum of the absolute values of the second partial derivatives of the spline. Analogous to the univariate case we introduce an alternative variational principle based on the integral of the square root of the sum of squares of the second partial derivatives of the bivariate spline. Whereas the Lavery integral in the bivariate case is not rotationally invariant, the alternative principle is. The alternative bivariate functional is shown to be a convex functional and, when expressed in terms of the partial slopes at the vertices of an underlying element, it is a convex function of these slopes. We have also shown that a rectangular strip bent only in the strip direction cannot be represented by means of a triangular rHTC element.

Analogous to the alternative weighted least squares algorithm introduced to compute the approximate univariate splines we introduce an alternative weighted least squares algorithm in the bivariate case. Although we have not introduced a convergence analysis of the alternative algorithms in this paper, computational experience has shown that the iterative, Gauss-Seidel based, algorithm used provides qualitatively satisfactory results in only a few iterations of the main loop.
